# Comparison of Synthetic Data Generation Techniques for Control Group Survival Data in Oncology Clinical Trials: Simulation Study

**DOI:** 10.2196/55118

**Published:** 2024-06-18

**Authors:** Ippei Akiya, Takuma Ishihara, Keiichi Yamamoto

**Affiliations:** 1 Biometrics, ICON Clinical Research GK, Tokyo, Japan; 2 Innovative and Clinical Research Promotion Center, Gifu University Hospital, Gifu, Japan; 3 Division of Data Science, Center for Industrial Research and Innovation, Translational Research Institute for Medical Innovation, Osaka Dental University, Osaka, Japan

**Keywords:** oncology clinical trial, survival analysis, synthetic patient data, machine learning, SPD, simulation

## Abstract

**Background:**

Synthetic patient data (SPD) generation for survival analysis in oncology trials holds significant potential for accelerating clinical development. Various machine learning methods, including classification and regression trees (CART), random forest (RF), Bayesian network (BN), and conditional tabular generative adversarial network (CTGAN), have been used for this purpose, but their performance in reflecting actual patient survival data remains under investigation.

**Objective:**

The aim of this study was to determine the most suitable SPD generation method for oncology trials, specifically focusing on both progression-free survival (PFS) and overall survival (OS), which are the primary evaluation end points in oncology trials. To achieve this goal, we conducted a comparative simulation of 4 generation methods, including CART, RF, BN, and the CTGAN, and the performance of each method was evaluated.

**Methods:**

Using multiple clinical trial data sets, 1000 data sets were generated by using each method for each clinical trial data set and evaluated as follows: (1) median survival time (MST) of PFS and OS; (2) hazard ratio distance (HRD), which indicates the similarity between the actual survival function and a synthetic survival function; and (3) visual analysis of Kaplan-Meier (KM) plots. Each method’s ability to mimic the statistical properties of real patient data was evaluated from these multiple angles.

**Results:**

In most simulation cases, CART demonstrated the high percentages of MSTs for synthetic data falling within the 95% CI range of the MST of the actual data. These percentages ranged from 88.8% to 98.0% for PFS and from 60.8% to 96.1% for OS. In the evaluation of HRD, CART revealed that HRD values were concentrated at approximately 0.9. Conversely, for the other methods, no consistent trend was observed for either PFS or OS. CART demonstrated better similarity than RF, in that CART caused overfitting and RF (a kind of ensemble learning approach) prevented it. In SPD generation, the statistical properties close to the actual data should be the focus, not a well-generalized prediction model. Both the BN and CTGAN methods cannot accurately reflect the statistical properties of the actual data because small data sets are not suitable.

**Conclusions:**

As a method for generating SPD for survival data from small data sets, such as clinical trial data, CART demonstrated to be the most effective method compared to RF, BN, and CTGAN. Additionally, it is possible to improve CART-based generation methods by incorporating feature engineering and other methods in future work.

## Introduction

When submitting an application for the approval of a new pharmaceutical product to health authorities, it is imperative to demonstrate its efficacy and safety through multiple clinical trials. However, 86% of these trials encounter difficulties meeting the targeted number of subjects within the designated recruitment period, often leading to extensions of the trial duration or completion of the trial without reaching the target number of subjects [1-3]. The challenge of patient recruitment not only delays the submission of regulatory applications but also hinders the timely provision of effective treatment to patients, which consequently contributes to increased development costs and the escalation of drug prices and potentially exacerbates the strain on health care financing.

In recent years, the use of real-world data (RWD) has emerged as a potential solution for addressing these issues. The Food and Drug Administration has also released draft guidelines [4], garnering attention on the application of RWD as an external control arm in clinical trials [5,6]. Furthermore, it has been reported that it is possible to optimize eligibility using RWD and machine learning, thereby increasing the number of eligible subjects that can be included [7].

In addition to these approaches, we hypothesize that it is possible to generate synthetic patient data (SPD) from control arm data in past clinical trials and use it to establish a control arm for a new clinical trial. The use of SPD, an emerging research approach in the health care research field [8-17], involves the generation of fictitious individual patient-level data from real data, which possess statistical properties similar to those of actual data. This approach is anticipated to facilitate health care research while addressing data privacy concerns [14,18-21].

Regarding its application in clinical trials, concerns have been raised about the feasibility of generating SPDs with statistical properties similar to those of actual data due to the relatively smaller volume of clinical trial data compared to RWD, such as electronic health records or registry data. However, previous studies [22-25] have reported the successful generation of SPDs with statistical properties generally comparable to the actual data, although there are certain limitations. Additionally, with the expansion of clinical trial data-sharing platforms such as ClinicalStudyDataRequest.com, Project Data Sphere, and Vivli, acquiring subject-level clinical trial data has become more accessible. Consequently, advancements in research on the utility of SPD and the expansion of clinical trial data-sharing platforms are expected to have potential applications in clinical trials.

Our focus lies in the application of this technology in oncology clinical trials that evaluate popular efficacy end points such as overall survival (OS) and progression-free survival (PFS)–related survival functions and median survival time (MST) [26]. In previous studies on SPD, there has been a notable emphasis on reporting patient background data and single–time point data [22-25]. However, research focusing specifically on the relationship between SPD and survival data remains relatively insufficient [27].

As the first step in examining our hypothesis that the use of SPD can be beneficial in accelerating health care research, the aim of this study was to determine the most suitable SPD generation method for oncology trials, specifically focusing on both OS and PFS, which are set as the primary evaluation end points in oncology trials. To achieve this goal, we conducted a comparative simulation of 4 generation methods: classification and regression trees (CART) [28], random forest (RF) [29], Bayesian network (BN) [30], and the conditional tabular generative adversarial network (CTGAN) approach [31], and the performance of each method was evaluated.

## Methods

### Overview

To generate the SPD, subject-level clinical trial data were obtained from Project Data Sphere for the following 4 clinical trials (Table 1): (1) each had a different cancer type, (2) included control arm data, (3) contained both OS and PFS data, and (4) had a ready data format for analysis.

**Table 1.** List of selected oncology clinical trials in this study.

ClinicalTrials.gov ID	Titles	Phase	Cancer type	Intervention for the control arm	Subjects in the control arm, n
NCT00119613	A Randomized, Double-Blind, Placebo-Controlled Study of Subjects With Previously Untreated Extensive-Stage Small-Cell Lung Cancer (SCLC) Treated With Platinum Plus Etoposide Chemotherapy With or Without Darbepoetin Alfa.	III	Small cell lung cancer	Placebo	232
NCT00339183	A Randomized, Multicenter Phase 3 Study to Compare the Efficacy of Panitumumab in Combination With Chemotherapy to the Efficacy of Chemotherapy Alone in Patients With Previously Treated Metastatic Colorectal Cancer.	III	Metastatic colorectal cancer	FOLFIRI^a^ Alone	476
NCT00339183	A Phase 3 Randomized Trial of Chemotherapy With or Without Panitumumab in Patients With Metastatic and/or Recurrent Squamous Cell Carcinoma of the Head and Neck (SCCHN).	III	Recurrent or metastatic (or both) head and neck cancer	Cisplatin and 5-fluorouracil	260
NCT00703326	A Multicenter, Multinational, Randomized, Double-Blind, Phase III Study of IMC-1121B Plus Docetaxel versus Placebo Plus Docetaxel in Previously Untreated Patients With HER2-Negative, Unresectable, Locally-Recurrent or Metastatic Breast Cancer.	III	Breast cancer	Placebo and docetaxel	382

a FOLFIRI: panitumumab plus fluorouracil, leucovorin, and irinotecan.

### Preparation of the Training Data Set

The patient data for the control arm contained within each trial data set were extracted and used as the actual data for the training data set. The selection of variables in the training data set aimed to include as many variables related to the subjects’ background as possible, excluding variables concerning tests and evaluations conducted during the trials. Furthermore, variables that had the same value were excluded, even if they were related to the subjects’ background (Multimedia Appendices 1-4).

### Generation of Synthetic Data

The SPDs in this study were generated using the following 4 methods:

1. CART: the synthpop package (version 1.8) in R (The R Foundation) was used, specifying the cart method for the syn function’s method argument.
2. RF: the synthpop package (version 1.8) in R was used, specifying the Ranger method for the syn function’s method argument.
3. BN: the bnlearn package (version 4.9) in R was used to conduct structural learning through the score-based algorithm hill-climbing, followed by parameter estimation using the bn.fit function. The default maximum likelihood estimator was used for parameter estimation.
4. CTGAN: the CTGANSynthesizer module included in the Python package sdv (version 1.3) was used.

In all these generation methods, to ensure the absence of conflicting data regarding the relationship between PFS and OS, constraints were set to ensure that the values of PFS and OS were greater than zero and that PFS was less than or equal to OS. Specific individual patient data in the generated SPD, which did not meet these constraints, were excluded, and new individual patient data were regenerated. The SPDs were generated in a manner that equaled the number of subject-level data to the record count in the actual data.

To ensure the reproducibility of SPD generation, 1000 random numbers were generated as seed values using the Mersenne Twister algorithm. The same seed value set was used for all generation methods.

### Statistical Analysis

#### Histogram

Histograms were created to visually inspect the distributions of the MST of the synthetic data (MSTS) for PFS and OS for the 1000 SPD data sets generated by each method. The histograms also included the MST of the actual data (MSTA) as a vertical line and the range of its 95% CI as a rectangular background. For PFS and OS, a higher percentage of MSTS covered by the 95% CI of the MSTA was determined to indicate a greater level of reliability for the generation method.

#### Evaluation of Similarity

A hazard ratio (HR) of 1 signifies that the 2 survival functions are entirely identical. Thus, the closer the HR is to 1, the more similar the 2 survival functions are. Accordingly, based on the following calculation formula, the HR distance (HRD) for PFS and OS from the SPD and the actual data were computed and evaluated:


HRD=1−abs(HR−1)

#### Kaplan-Meier Plot

In the evaluation of similarity, the SPD that showed the highest HRD value was considered the best case, and the SPD with the lowest HRD value was considered the worst case. Three groups of Kaplan-Meier (KM) plots were created, including the actual data, the best case, and the worst case for each SPD generation method. The best case and worst case for each SPD generation method in both PFS and OS were compared to actual survival by using the log rank test. Multiple comparisons were not performed, nor were *P* values adjusted because controlling for the type I error rate does not affect the conclusions of this study.

Since the purpose of this study was to evaluate the method of generating SPD that closely resemble actual survival data, it might be unnecessary to calculate a *P* value that indicates a significant difference from actual survival, but the *P* value was calculated in this study from the viewpoint that if a significant difference is also observed in the best-case, that method should not be adopted.

All analyses and data generation were performed using R (version 4.3.1; The R Foundation) and Python (version 3.10; Python Software Foundation).

### Ethical Considerations

Ethical review was not needed for this simulation study for methodology comparison. All actual clinical trial data sets obtained from Project Data Sphere were used in accordance with relevant guidelines and regulations when the clinical trials were conducted.

## Results

**Figure 1.**
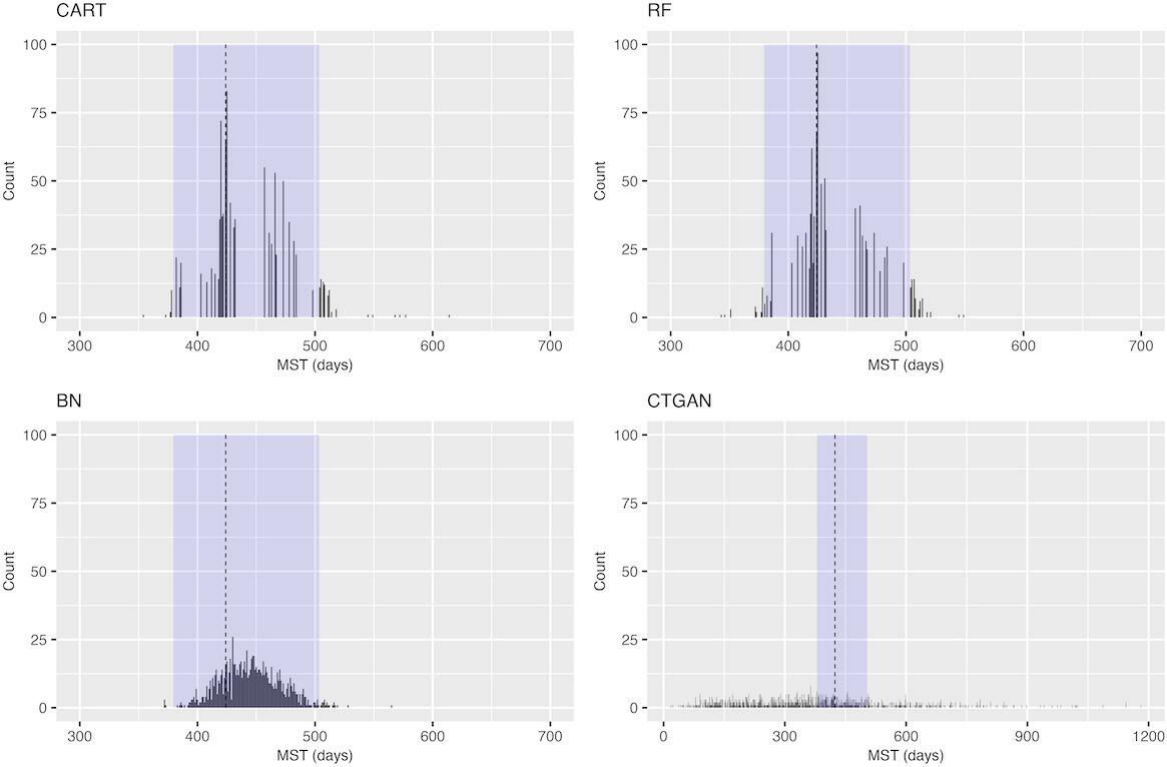
Histogram of the median survival time of the synthetic data for progression-free survival in the NCT00703326 trial. The dashed vertical line represents the median survival time of the actual data, and the light blue background indicates its 95% CI. BN: Bayesian network; CART: classification and regression tree; CTGAN: conditional tabular generative adversarial network; MST: median survival time; RF: random forest.

Figure 1 shows a histogram of the MSTS for PFS in the NCT00703326 trial. Using CART, RF, and BN, most of the generated MSTS values were within the 95% CI of the MSTA. In contrast, when CTGAN was used, SPD generation resulted in a widened variance in the distribution of MSTS. For the MSTS of PFS in the other 3 trials, RF exhibited a shift in the distribution of the MSTS, shortening the survival period, while BN displayed a shift in the distribution and prolonged the survival period. Similar trends to Figure 1 were observed for CART and CTGAN (Multimedia Appendices 5-7).

**Figure 2.**
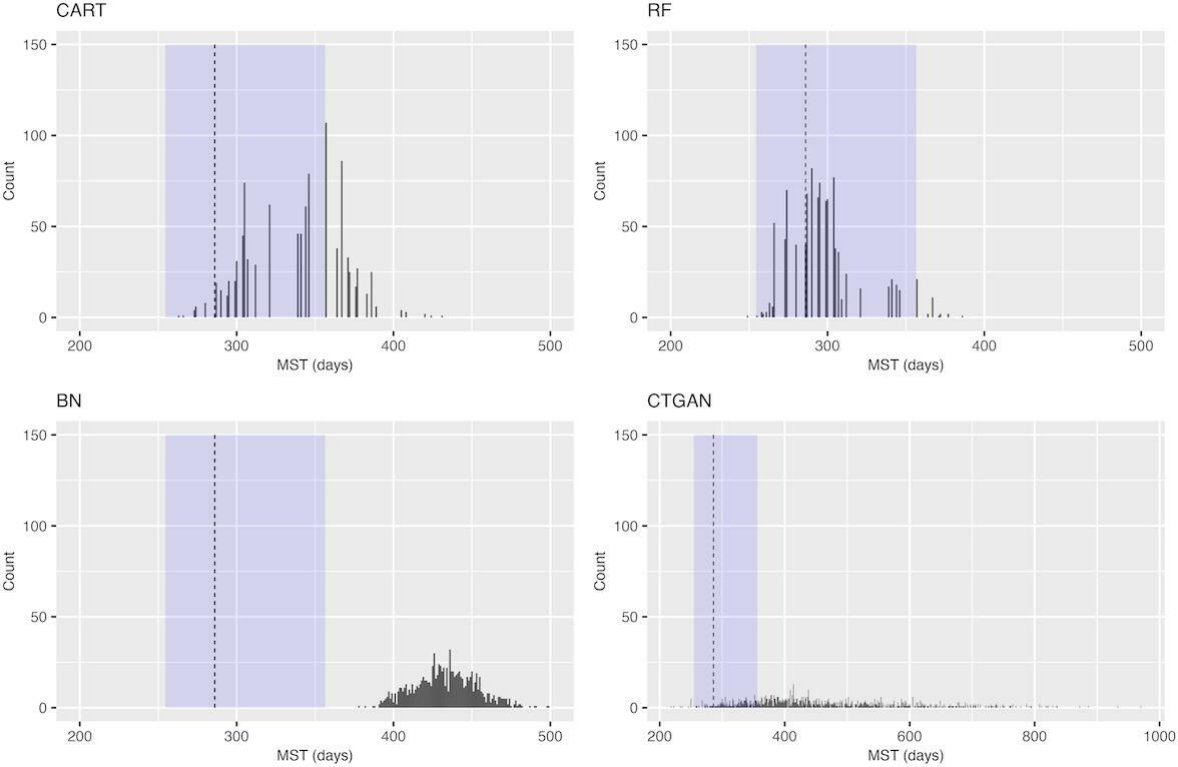
Histogram of the median survival time of the synthetic data of overall survival in the NCT00460265 trial. The dashed vertical line represents the median survival time of the actual data, and the light blue background indicates its 95% CI. BN: Bayesian network; CART: classification and regression tree; CTGAN: conditional tabular generative adversarial network; MST: median survival time; RF: random forest.

Figure 2 displays a histogram of the MSTS for OS in the NCT00460265 trial. The divergence from the PFS findings is that the MSTS of RF was more frequently included within the 95% CI of the MSTA, with similar results observed in other trials (Multimedia Appendices 8-10). In other aspects, similar findings were obtained as with the PFS.

Table 2 presents the number and proportion of the generated MSTS values included within the 95% CI of the MSTA for each trial and each method. In the case of CART for PFS, a high percentage ranging from 88.8% to 98.1% was exhibited for all trials. However, the OS ranged from 60.8% to 96.1%, with some trials displaying a lower percentage than the PFS.

**Table 2.** The number and proportion of median survival times of the synthetic data (MSTSs) falling within the 95% CI of the median survival time of the actual data (MSTA).

		ClinicalTrials.gov ID			
		NCT00119613	NCT00339183	NCT00460265	NCT00703326
**Progression-free survival**					
	MSTA (95% CI)	169 (163-183)	155 (121-168)	133 (121-167)	424 (380-504)
**MSTSs, n (%)**					
	CART^a^ (n=1000)	981 (98.1)	888 (88.8)	955 (95.5)	918 (91.8)
	RF^b^ (n=1000)	693 (69.3)	248 (24.8)	426 (42.6)	919 (91.9)
	BN^c^ (n=1000)	10 (1.0)	0 (0.0)	37 (3.7)	976 (97.6)
	CTGAN^d^ (n=1000)	65 (6.5)	378 (37.8)	322 (32.2)	254 (25.5)
**Overall survival**					
	MSTA (95% CI)	276 (259-303)	361 (319-393)	286 (255-357)	1452 (1417-1507)
**MSTSs, n (%)**					
	CART (n=1000)	831 (83.1)	608 (60.8)	719 (71.9)	961 (96.1)
	RF (n=1000)	757 (75.7)	697 (69.7)	980 (98.0)	599 (59.9)
	BN (n=1000)	0 (0.0)	0 (0.0)	0 (0.0)	622 (62.2)
	CTGAN (n=1000)	72 (7.2)	155 (15.5)	197 (19.7)	81 (8.5)

a CART: classification and regression tree.

b RF: random forest.

c BN: Bayesian network.

d CTGAN: conditional tabular generative adversarial network.

For RF, a high proportion of 91.9% was observed for PFS in the NCT00703326 trial and 98.0% for OS in the NCT00460265 trial, whereas in other cases, the proportion for RF was not as high as that for CART.

In the case of BN, proportions of 97.6% and 62.2% were observed for PFS and OS, respectively, in the NCT00703326 trial, but in the other 3 trials, BN showed an extremely low percentage ranging from proportion ranging from 0.0% to 3.7%.

CTGAN showed a low proportion ranging from 6.5% to 37.8% for both PFS and OS in all trials.

**Figure 3.**
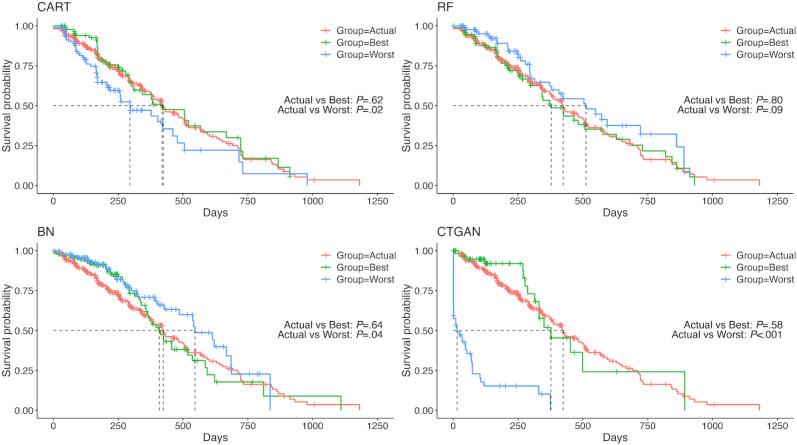
Kaplan-Meier plots for progression-free survival in the NCT00703326 trial. BN: Bayesian network; CART: classification and regression tree; CTGAN: conditional tabular generative adversarial network; RF: random forest.

Figure 3 shows the KM plot for PFS in the NCT00703326 trial. The best-case curves of CART and RF were similar to the actual data curve. In contrast, for BN and CTGAN, even the best-case curves deviated from the actual data curve. In other trials, some SPD did not show a similar trend. However, at least for the best-case scenarios of CART and RF, the generated synthetic survival curves closely resembled the actual survival curve (Multimedia Appendices 11-13).

**Figure 4.**
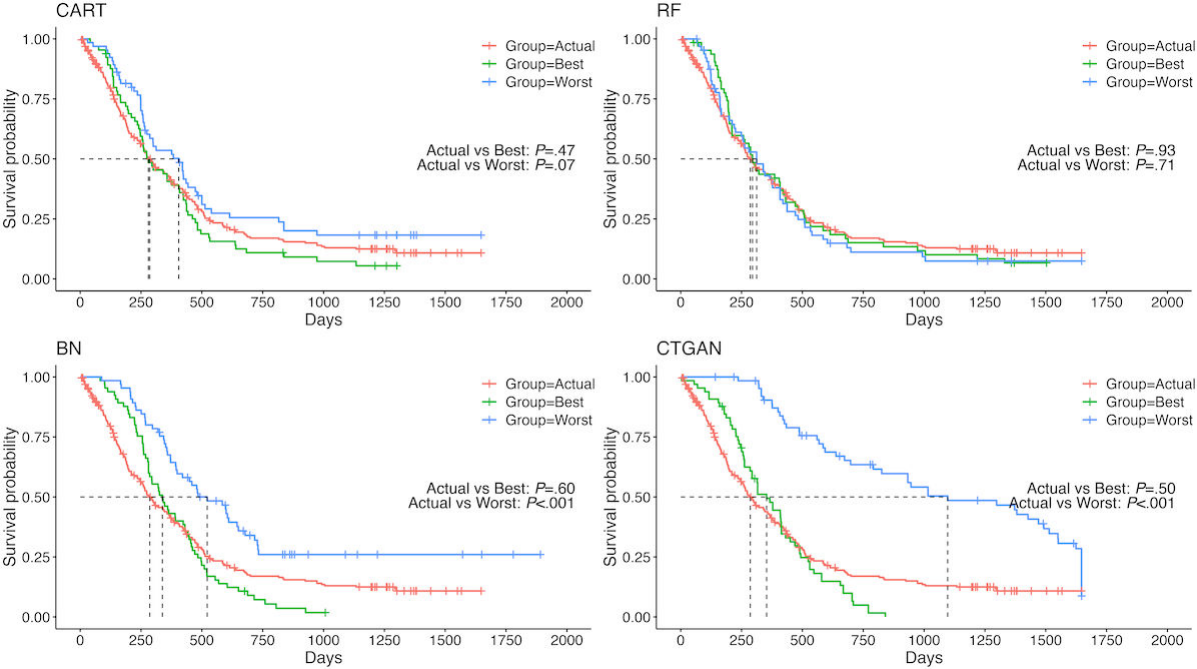
Kaplan-Meier plots for overall survival in the NCT00460265 trial. BN: Bayesian network; CART: classification and regression tree; CTGAN: conditional tabular generative adversarial network; RF: random forest.

Figure 4 displays the KM plot for OS in the NCT00460265 trial. Similar to the KM plots for PFS, the best-case curves of CART and RF resembled the actual data curve, whereas those of BN and CTGAN deviated from the actual data curve. These trends were also observed in other trials (Multimedia Appendices 14-16).

**Figure 5.**
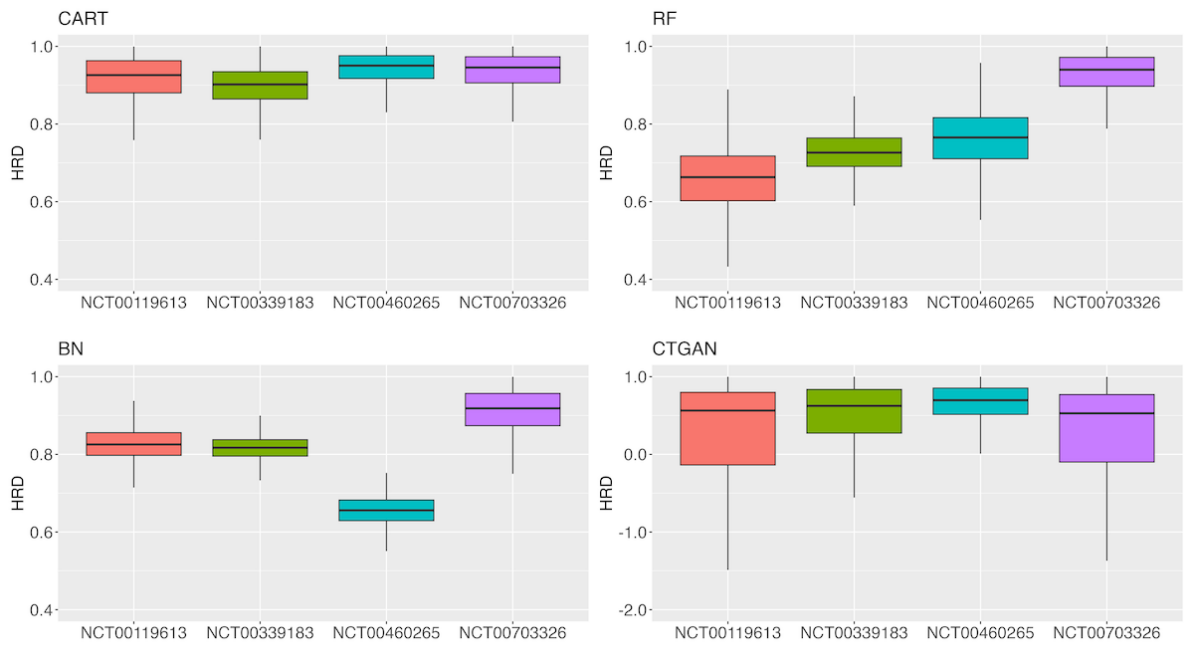
Box plot of progression-free survival hazard ratio distance (HRD) for each method and clinical trial. BN: Bayesian network; CART: classification and regression tree; CTGAN: conditional tabular generative adversarial network; RF: random forest.

Figures5  6 present box plots of the HRD. When using CART, the HRD values for both PFS and OS in all trials were concentrated at approximately 0.9. Conversely, for the other methods, no consistent trend was observed for either PFS or OS.

**Figure 6.**
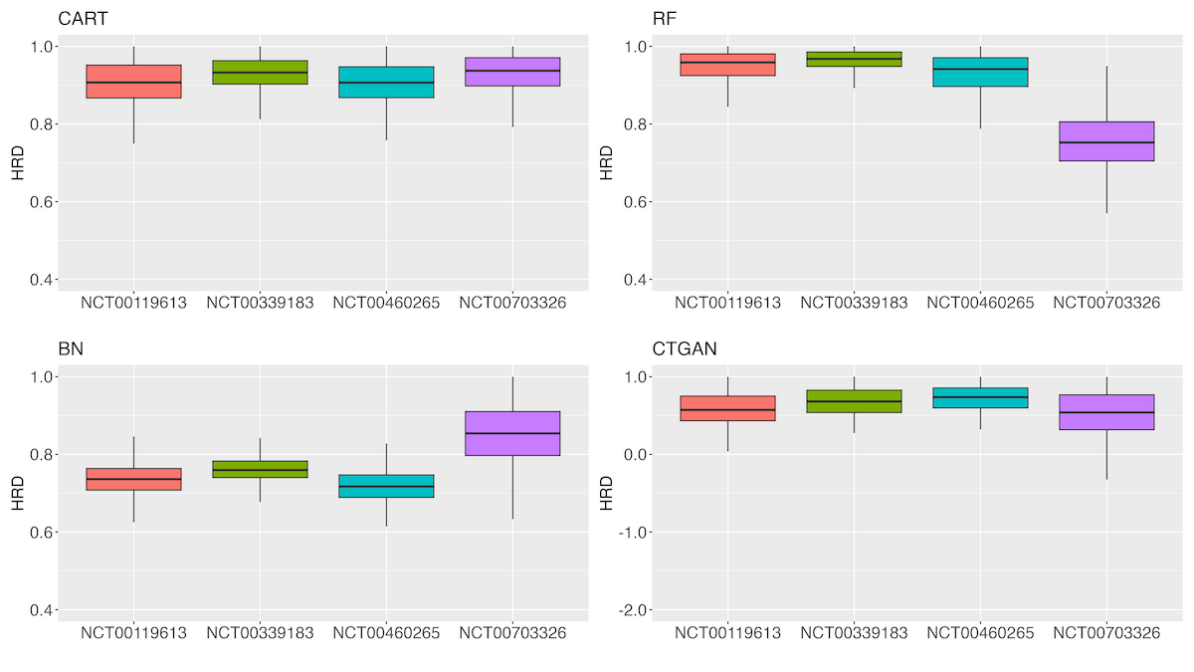
Box plot of overall survival hazard ratio distance (HRD) for each method and clinical trial. BN: Bayesian network; CART: classification and regression tree; CTGAN: conditional tabular generative adversarial network; RF: random forest.

## Discussion

Regarding the survival SPD, CART often yielded better results than the other methods in evaluations using MST, HRD, and visual analysis via KM plots. Given the crucial importance of the hazard ratio and MST as end points in oncology trials [26], demonstrating the utility of both of these evaluation metrics is essential. Therefore, using CART for generating survival SPD was suggested as a beneficial approach.

While both CART and RF generally yielded preferable results across all trials, they share the common characteristic of using tree models. RF, with its use of the bootstrap method for resampling and constructing tree models for ensemble learning, is known to prevent overfitting. In general, in terms of constructing machine learning models with high generalization performance, RF performs better than CART. However, CART is prone to overfitting as the layers of the tree become deeper [32]. Although RF is considered a superior method for constructing high–generalization-performance machine learning models, the results from Table 2 and the KM plots in this study suggest that CART is a better approach than RF. This discrepancy might be due to differing views on what is a higher performance between the machine learning prediction model and SPD. In the machine learning prediction model, it is important to prevent overfitting and reduce bias; however, SPD is expected to match its statistical properties with actual data. Thus, in the case of SPD, the overfitting suppression mechanism possessed by RF might have resulted in inferiority to that of CART from the perspective of improving similarity.

In the case of using BN, the percentage of MSTSs falling within the 95% CI of MSTAs was 0% for the PFS of the NCT00339183 trial, and for OS, this phenomenon also occurred in the NCT00119613, NCT00339183, and NCT0046265 trials. This implies that the SPD failed to accurately reflect the statistical properties of the actual data. Conversely, a high value of 97.6% was observed for the PFS in the NCT00703326 trial. The reason for this discrepancy could not be determined on the basis of the results of this study. Tucker et al [24] reported that they could generate data highly similar to actual data when using BN for the generation of SPD, which differs from our findings. One notable difference is that while Tucker et al [24] used a large-scale actual data set of 27.5 million patients for their study, this study used only a few hundred patients for training data. This difference likely had a significant impact on the accuracy of the SPD generation model, resulting in conflicting results. However, the SPD generated by BN were not distributed in the direction of shortening PFS or OS. Thus, this would not be harmful when the SPD generated by BN is used as a more conservative control arm in clinical trials.

Using CTGAN, the percentage of the MSTSs falling within the 95% CI of the actual data was low, indicating low performance associated with the generation of SPD that reflect the statistical properties of the actual data. However, Krenmayr et al [23] reported favorable performance results when using the same generative adversarial network (GAN)–based methods and RWD. The differences between their study and our study were as follows: their study did not include SPD on survival time or generate multiple SPD data sets from the same actual data, and there was a large amount of individual patient data in their study. In particular, focusing on the amount of individual patient data, the number of patients in each trial included in this study was relatively small, with the NCT00119613 trial having 232 patients, the NCT00339183 trial having 476 patients, the NCT0046265 trial having 260 patients, and the NCT00703326 trial having 382 patients, while the trial conducted by Krenmayr et al [23] had 500 or more patients. GAN-based methods using deep neural networks are known to perform poorly with small amounts of data [25,33]. In this study, although the NCT00339183 trial had the largest number of individual patient data, the best case of CTGAN for NCT00339183 produced a KM plot similar to the actual data, suggesting that a larger data set yields better results. Thus, there is no contradiction. Another characteristic of using CTGAN in this study was the larger variance in the estimated MSTSs, as indicated in Figures 1 and 2. Goncalves et al [34] showed that using MC-MedGAN, a GAN-based method, to generate an SPD from small data resulted in a large SD of the data utility metrics, leading to results with larger variance, similar to those of this study. Therefore, it is extremely challenging to generate useful SPD by applying GAN-based methods to small data sets, such as clinical trial data.

When generating SPDs for survival data and using them as a certain arm in a clinical trial, it is important to verify that the statistical properties closely match those of the actual data with the MST and the hazard ratio with the actual data being close to 1. Based on our results, we conclude that CART, which can concentrate the MSTSs within the range of 95% CI of MSTAs and approximately 0.9 for HRD, is an efficient method for generating SPD that meets the abovementioned conditions. However, even when using CART, slight variations were observed in the MSTSs, and some cases fell outside the 95% CI of the MSTAs, as revealed by our results. Therefore, for practical use, it is necessary to verify that the MSTSs are included in the 95% CI of the MSTAs and that both are close in value. It is also necessary to verify whether the HRD of the actual data and the SPD are close to 1 and then decide whether to adopt the generated SPD. Hence, the generation process must be repeated until an acceptable SPD is obtained. There may also be a need to use statistical methods to match characteristics between the SPD and the actual treatment arm in clinical trials.

In this study, even the most useful CART method produced SPDs that did not meet the requirements of MST and HRD. We expect that this issue will be addressed by incorporating feature engineering, such as dimension reduction, imputing missing values, derived variable creation, and other processing. Additionally, in clinical research, as subgroup analyses are frequently conducted, it is necessary to improve the generation method to reflect the statistical properties of the actual data even when the data are divided into subgroups under certain conditions. Moreover, from the perspective of data privacy, it is essential to incorporate approaches to prevent data reidentification into the generation method [35].

In conclusion, as a method for generating SPD for survival data from small data sets, such as clinical trial data, CART is the most effective method for generating SPD that meet the 2 conditions of having an MSTSs close to the MSTAs and an HRD close to 1. However, as SPD might be generated, which do not meet these 2 conditions, it is necessary to incorporate mechanisms to improve a CART-based generation method in future studies. Overcoming these challenges would make it possible to reduce the recruitment period and costs of clinical trial participants to ≥50% in comparative trials of new drug development against existing therapeutic drugs. This approach could accelerate clinical development, similar to the use of RWD.

## Supplementary material

10.2196/55118 Multimedia Appendix 1 Variables used to generate synthetic patient data from the NCT00119613 trial.

10.2196/55118 Multimedia Appendix 2 Variables used to generate synthetic patient data from the NCT00339183 trial.

10.2196/55118 Multimedia Appendix 3 Variables used to generate synthetic patient data from the NCT00460265 trial.

10.2196/55118 Multimedia Appendix 4 Variables used for generating synthetic patient data from the NCT00703326 trial.

10.2196/55118 Multimedia Appendix 5 Histogram of the median survival time of the synthetic data for progression-free survival in the NCT00119613 trial. The dashed vertical line represents the median survival time for the actual data, and the light blue background indicates its 95% CI.

10.2196/55118 Multimedia Appendix 6 Histogram of the median survival times for the synthetic data for progression-free survival in the NCT00339183 trial. The dashed vertical line represents the median survival time of the actual data, and the light blue background indicates its 95% CI.

10.2196/55118 Multimedia Appendix 7 Histogram of the median survival times of the synthetic data for progression-free survival in the NCT00460265 trial. The dashed vertical line represents the median survival time of the actual data, and the light blue background indicates its 95% CI.

10.2196/55118 Multimedia Appendix 8 Histogram of the median survival times of the synthetic data for overall survival in the NCT00119613 trial. The dashed vertical line represents the median survival time of the actual data, and the light blue background indicates its 95% CI.

10.2196/55118 Multimedia Appendix 9 Histogram of the median survival times of the synthetic data for overall survival in the NCT00339183 trial. The dashed vertical line represents the median survival time of the actual data, and the light blue background indicates its 95% CI.

10.2196/55118 Multimedia Appendix 10 Histogram of the median survival times of the synthetic data for overall survival in the NCT00703326 trial. The dashed vertical line represents the median survival time of the actual data, and the light blue background indicates its 95% CI.

10.2196/55118 Multimedia Appendix 11 Kaplan-Meier plots for progression-free survival in the NCT00119613 trial.

10.2196/55118 Multimedia Appendix 12 Kaplan-Meier plots for progression-free survival in the NCT00339183 trial.

10.2196/55118 Multimedia Appendix 13 Kaplan-Meier plots for progression-free survival in the NCT00460265 trial.

10.2196/55118 Multimedia Appendix 14 Kaplan-Meier plots for overall survival in the NCT00119613 trial.

10.2196/55118 Multimedia Appendix 15 Kaplan-Meier plots for overall survival in the NCT00339183 trial.

10.2196/55118 Multimedia Appendix 16 Kaplan-Meier plots for overall survival in the NCT00703326 trial.

## Abbreviations

BN: Bayesian network
CART: classification and regression trees
CTGAN: conditional tabular generative adversarial network
GAN: generative adversarial network
HR: hazard ratio
HRD: hazard ratio distance
KM: Kaplan‒Meier
MST: median survival time
MSTA: median survival time of actual data
MSTS: median survival time of synthetic data
OS: overall survival
PFD: progression-free survival
RF: random forest
RWD: real-world data
SPD: synthetic patient data
