# Supplementary material for: Comparison of Synthetic Data Generation Techniques for Control Group Survival Data in Oncology Clinical Trials: Simulation Study
Source: JMIR Med Inform. 2024 Jun 18;12:e55118. doi: 10.2196/55118 (PMC11196245; doi:10.2196/55118)
Supplement: Multimedia Appendix 1 [file medinform-v12-e55118-s001.docx]

## Multimedia Appendix 1

Variables used to generate the SPD from NCT00119613

| Variable Name | Description |
| --- | --- |
| SEX | SEX |
| AGE | AGE |
| B_WGTN | Baseline Weight category |
| B_ECOGN | Baseline ECOG |
| B_LDHN | Baseline LDH |
| B_HGB | Baseline Hemoglobin (g/dL) |
| BSEPON | Baseline Serum EPO |
| MEDHINTN | History of Events of Historical Interest |
| PRTFN | RBC Tfn Before 1^st^ Dose of Study Drug |
| PFSDY | Time to Disease progression (days) |
| PFSCD | Disease Progression or Death? |
| DTHDY | Day of Death |
| DTH | Death? |
| B_HGB | Baseline Hemoglobin (g/dL) |
