# Supplementary material for: Comparison of Synthetic Data Generation Techniques for Control Group Survival Data in Oncology Clinical Trials: Simulation Study
Source: JMIR Med Inform. 2024 Jun 18;12:e55118. doi: 10.2196/55118 (PMC11196245; doi:10.2196/55118)
Supplement: Multimedia Appendix 2 [file medinform-v12-e55118-s002.docx]

## Multimedia Appendix 2

Variables used to generate the SPD from NCT00339183

| Variable Name | Description |
| --- | --- |
| AGE | Age in Years at Screening |
| SEX | Sex |
| RACCAT | Race Category |
| ATRT | Actual Treatment |
| PRBEV | Prior Bevacizumab? |
| PROXAL | Prior Oxaliplatin Exposure? |
| B_METACT | Number of BL Metas Site (cat) |
| B_LDHNM | Baseline LDH Value |
| BECOGICD | IVRS ECOG Performance Status Code |
| DIAGTYPE | Primary Tumor Diagnosis |
| PRADJYN | Prior Adjuvant Therapy? |
| LIVERMET | Metastases to Liver at Study Entry? |
| KRAS | KRAS Result |
| PFSDYCR | PFS Day (Central, RECIST) |
| PFSCR | PD on Study (Central, RECIST) or Death |
| DTHDY | Death Day |
| DTH | Death |
