# Supplementary material for: Comparison of Synthetic Data Generation Techniques for Control Group Survival Data in Oncology Clinical Trials: Simulation Study
Source: JMIR Med Inform. 2024 Jun 18;12:e55118. doi: 10.2196/55118 (PMC11196245; doi:10.2196/55118)
Supplement: Multimedia Appendix 3 [file medinform-v12-e55118-s003.docx]

## Multimedia Appendix 3

Variables used to generate the SPD from NCT00460265

| Variable Name | Description |
| --- | --- |
| AGE | Age in Years at Screening |
| SEX | Sex |
| RACE | Race Category |
| ATRT | Actual Treatment |
| PRHNTRTC | Prior Treatment for SCCHN CRF? |
| CARBOADD | Carboplatin >=1 doses? |
| CARBO2D | Carboplatin >=2 Doses? |
| B_ECOG | Baseline ECOG Performance Status |
| DIAGTYPE | Primary Tumor Diagnosis |
| PFSDYLRE | PFS Day |
| PFSLRE | PD on Study (Event Imp.) or Death |
| DTHDY | Death Day |
| DTH | Death |
