# Supplementary material for: Comparison of Synthetic Data Generation Techniques for Control Group Survival Data in Oncology Clinical Trials: Simulation Study
Source: JMIR Med Inform. 2024 Jun 18;12:e55118. doi: 10.2196/55118 (PMC11196245; doi:10.2196/55118)
Supplement: Multimedia Appendix 4 [file medinform-v12-e55118-s004.docx]

## Multimedia Appendix 4

Variables used for generating the SPD from NCT00703326

| Variable Name | Description |
| --- | --- |
| RACE | Race |
| PTAX | Strata 1: Prior Taxane Therapy |
| VMETA | Strata 2: Visceral Metastasis |
| HR | Strata 3: Hormone Receptor Status |
| GEOREG | Strata 4: Geographical Region |
| AGEGR1 | Pooled Age Group 1 |
| ECOGGR1 | Pooled ECOG Group 1 |
| REGST | Status at Registration |
| NBSTGR1 | Pooled Nb of Met Sites Group 1 |
| LESTYP | Lesion Type at Baseline |
| DFIGR1 | Pooled DFI Group 1 |
| ERSTGR1 | Pooled ER Group 1 |
| PRSTGR1 | Pooled PR Group 1 |
| TRPNEGFL | Triple Negative Flag |
| PHTXFL | Prior Hormonal Therapy Flag |
| PCTXFL | Prior Chemotherapy Flag |
| PACTFL | Prior Adjuvant/Neoadjuvant ACT Flag |
| PAFL | Prior Anthracycline Flag |
| PARCAT1 | Parameter Category 1 |
| AVAL | Analysis Value |
| CNSR | Censor |
| AVALCAT | Categorization of the Analysis Value |
