# Supplementary material for: Comparison of Synthetic Data Generation Techniques for Control Group Survival Data in Oncology Clinical Trials: Simulation Study
Source: JMIR Med Inform. 2024 Jun 18;12:e55118. doi: 10.2196/55118 (PMC11196245; doi:10.2196/55118)

## Multimedia Appendix 5

Histogram of the MSTSs for PFS in the NCT00119613 trial. The dashed vertical line represents the MSTA, and the light blue background indicates its 95% CI.


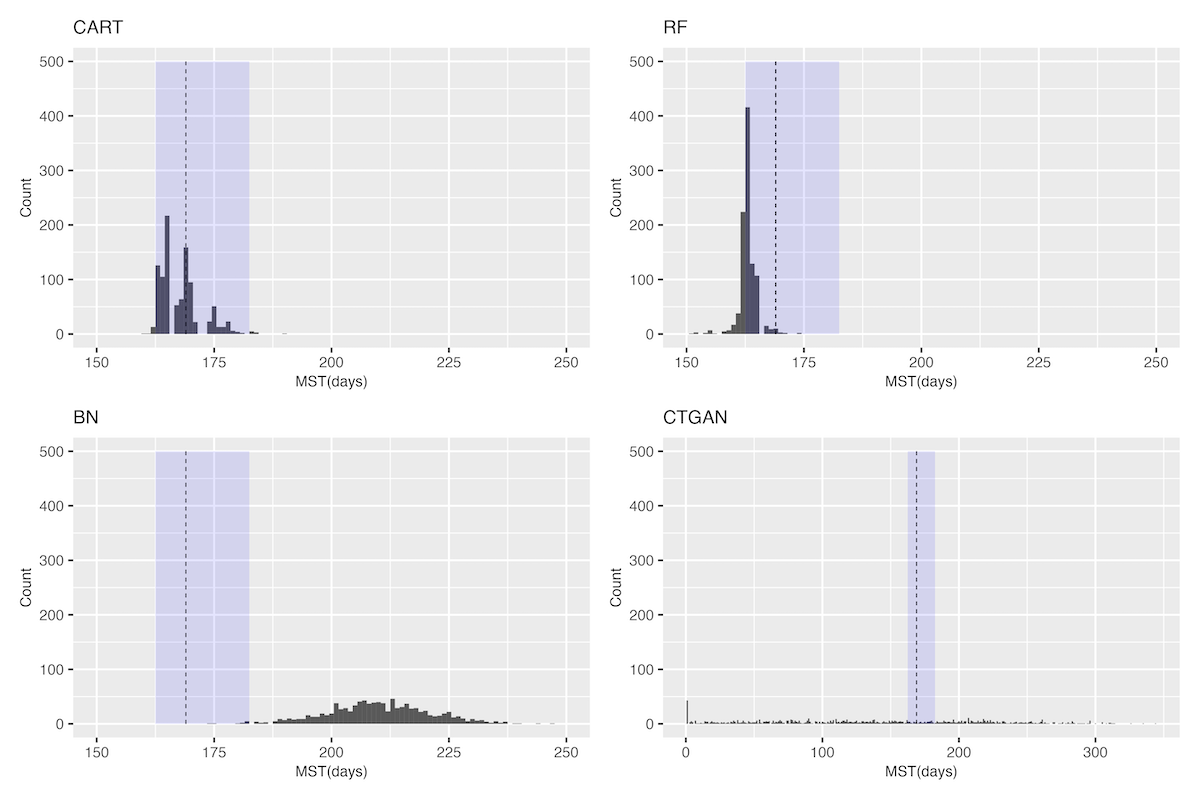

Supplement: Multimedia Appendix 5 [file medinform-v12-e55118-s005.docx]
