# Supplementary figures and images for: Comparison of Synthetic Data Generation Techniques for Control Group Survival Data in Oncology Clinical Trials: Simulation Study
Source: JMIR Med Inform. 2024 Jun 18;12:e55118. doi: 10.2196/55118 (PMC11196245; doi:10.2196/55118)

## Multimedia Appendix 11

KM plots for PFS in the NCT00119613 trial.


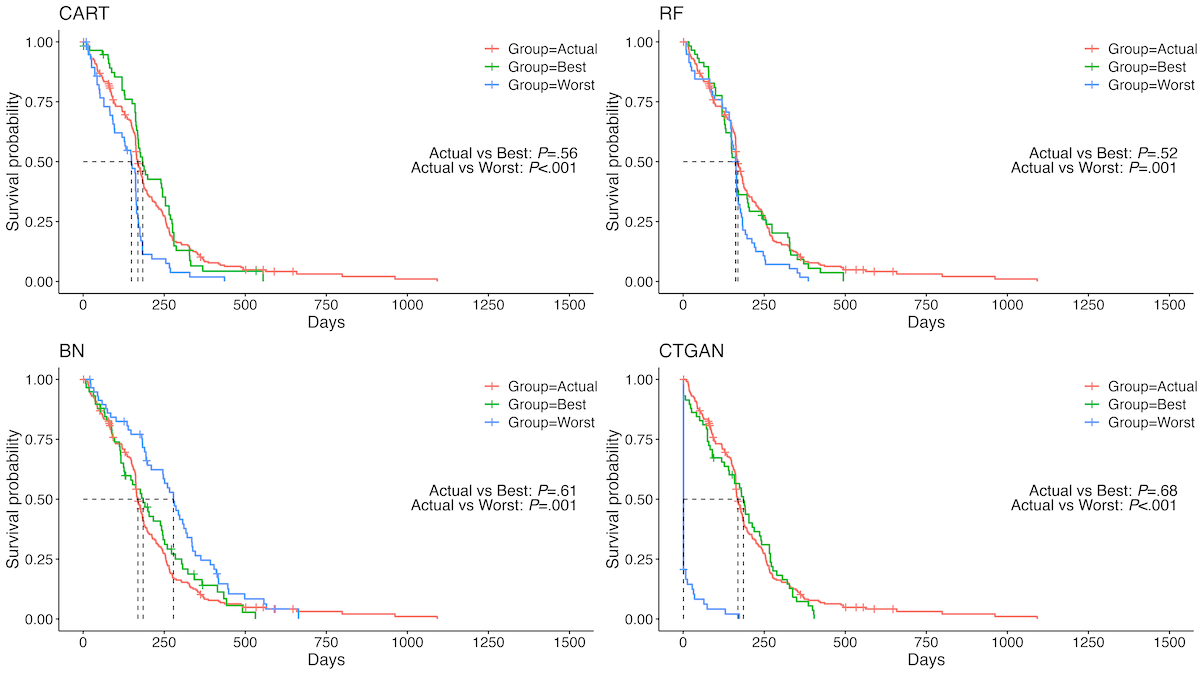

Supplement: Multimedia Appendix 11 [file medinform-v12-e55118-s011.docx]

## Multimedia Appendix 12

KM plots for PFS in the NCT00339183 trial.


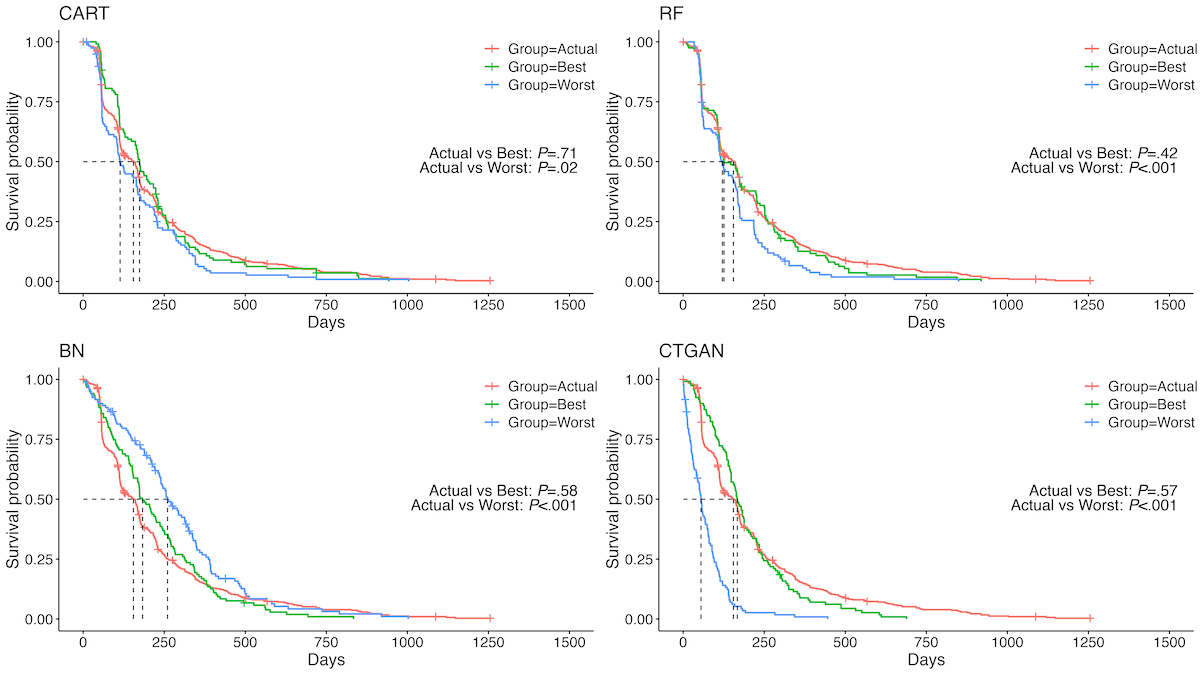

Supplement: Multimedia Appendix 12 [file medinform-v12-e55118-s012.docx]

## Multimedia Appendix 13

KM plots for PFS in the NCT00460265 trial.


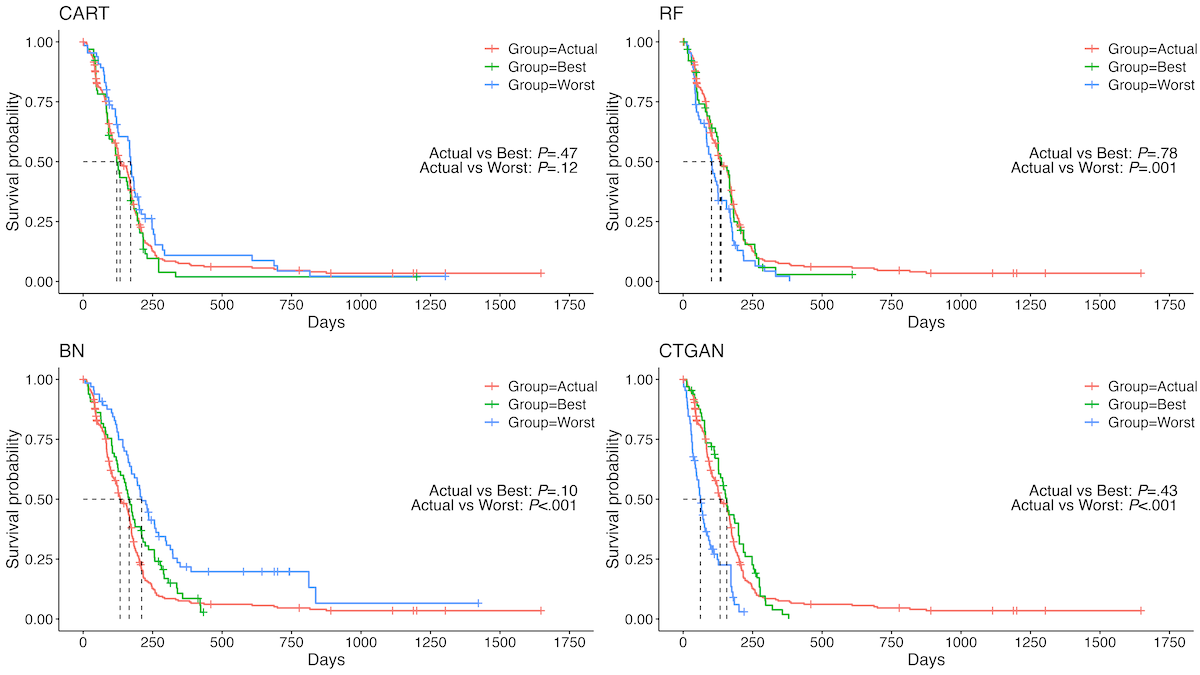

Supplement: Multimedia Appendix 13 [file medinform-v12-e55118-s013.docx]

## Multimedia Appendix 14

KM plots for OS in the NCT00119613 trial.


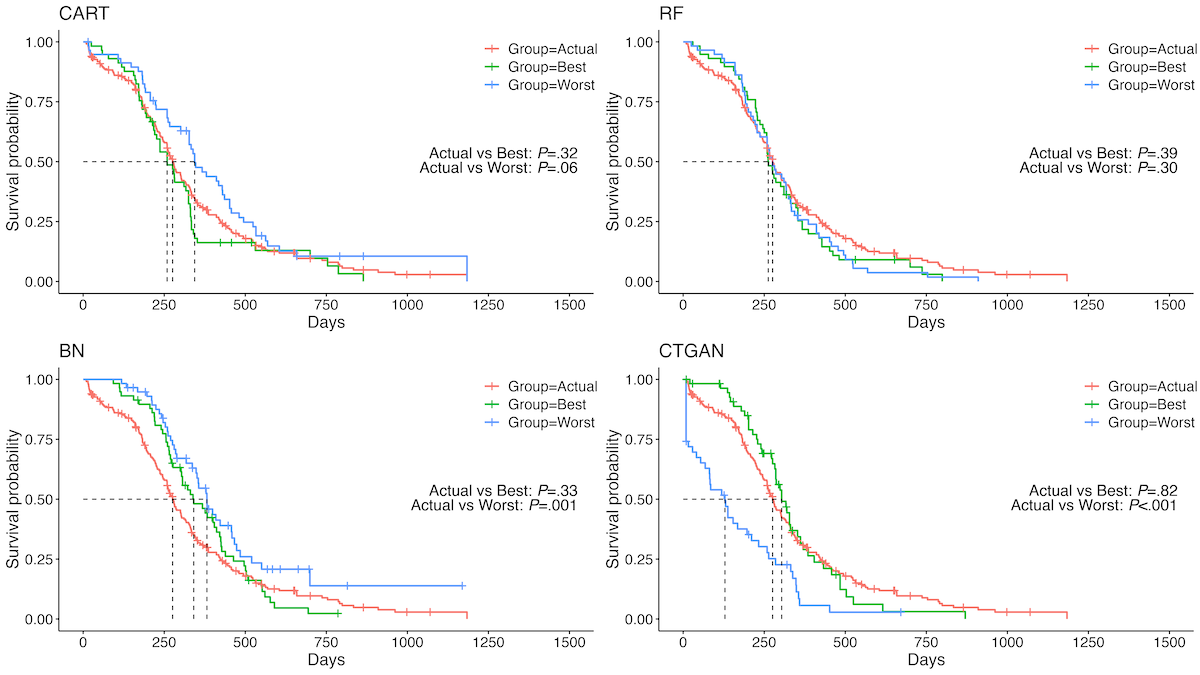

Supplement: Multimedia Appendix 14 [file medinform-v12-e55118-s014.docx]

## Multimedia Appendix 15

KM plots for OS in the NCT00339183 trial.


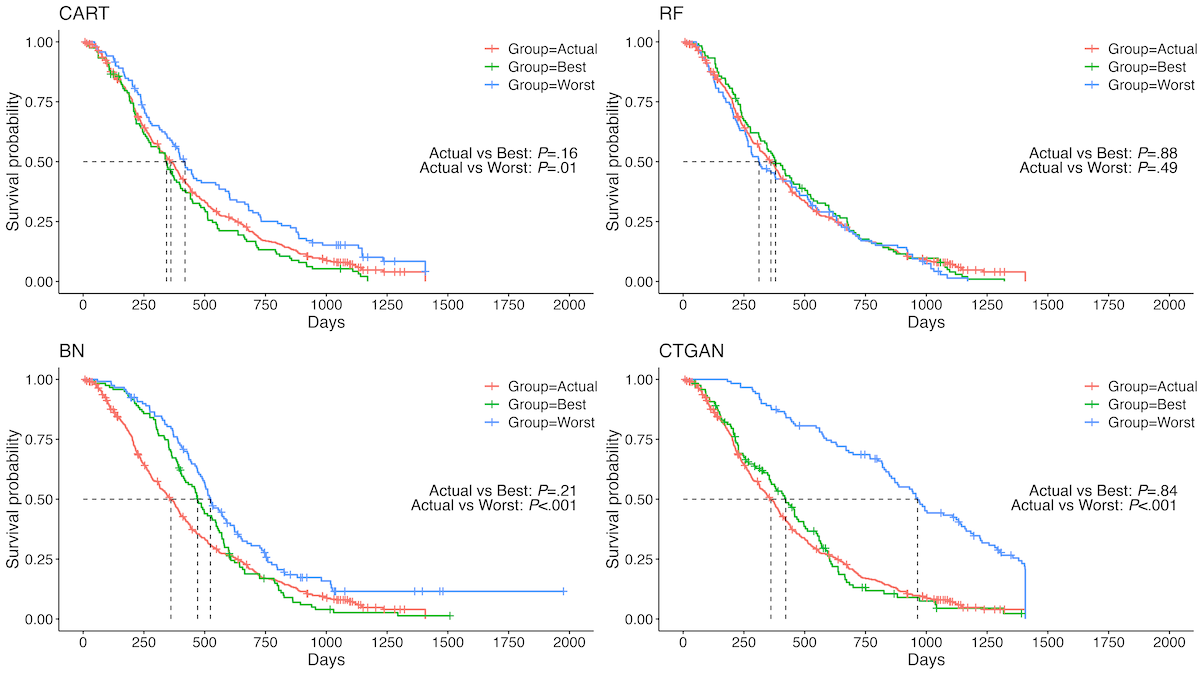

Supplement: Multimedia Appendix 15 [file medinform-v12-e55118-s015.docx]

## Multimedia Appendix 16

KM plots for OS in the NCT00703326 trial.


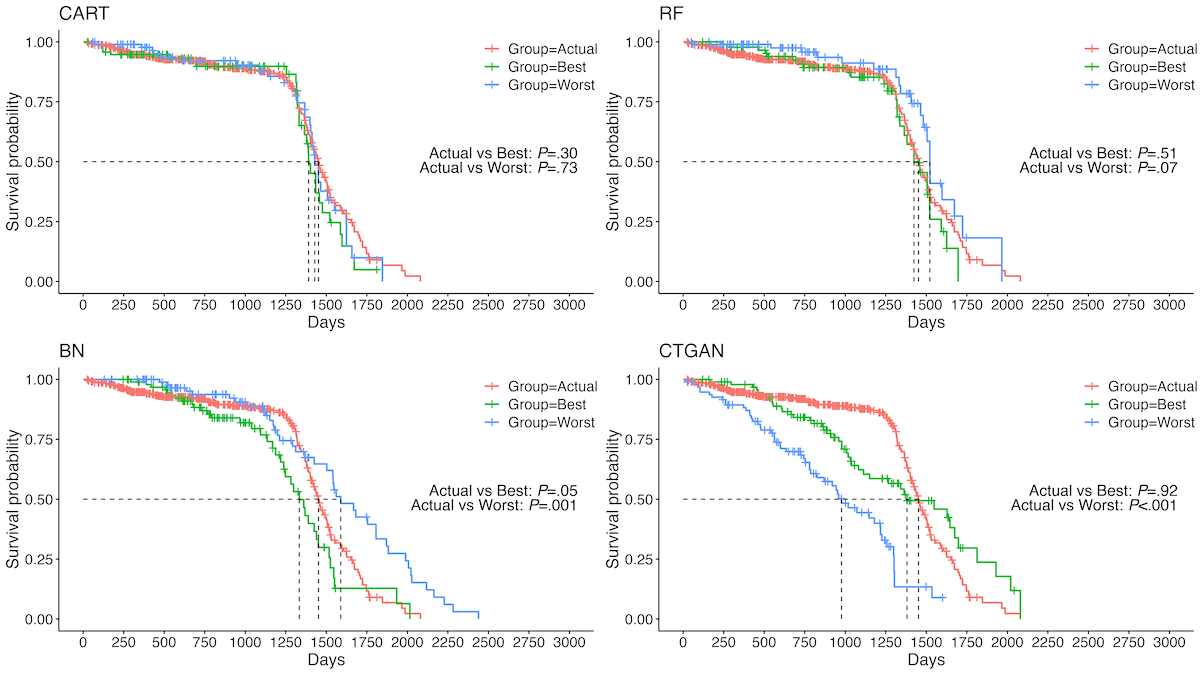

Supplement: Multimedia Appendix 16 [file medinform-v12-e55118-s016.docx]
